# Supplementary material for: Mechanism underlying delayed rectifying in human voltage-mediated activation Eag2 channel
Source: Nat Commun. 2023 Mar 16;14:1470. doi: 10.1038/s41467-023-37204-6 (PMC10020445; doi:10.1038/s41467-023-37204-6)
Supplement: Supplementary file 1 — Supplementary Information [file 41467_2023_37204_MOESM1_ESM.pdf]

Supplementary Information for Mechanism underlying delayed rectifying in human voltage-mediated activation Eag2 channel

Mingfeng Zhang[1,2,#], Yuanyue Shan[1,2,#], Duanqing Pei[2*]

Corresponding to : peiduanqing@westlake.edu.cn

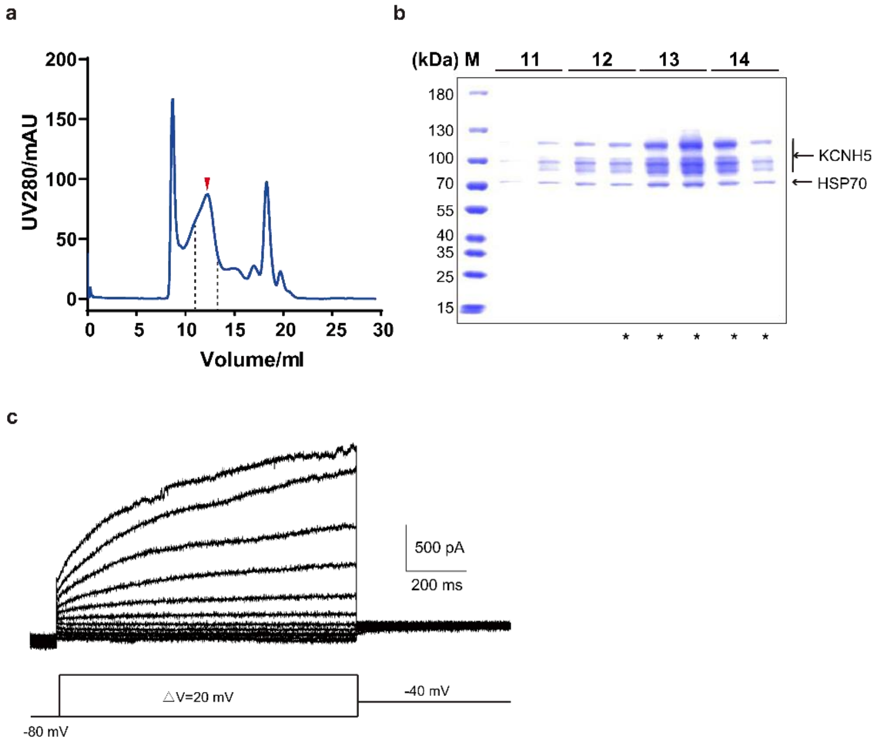

Supplementary Fig. 1 Purification of the hEag2 channel. a, Representative size-exclusion chromatography (SEC) trace of purified hEag2 channel. **b,** SDS-PAGE gel of peak fractions stained with Coomassie blue. The predicted molecular weight based on protein sequence for a single hEag2 subunit is 111.89 kDa. Peak fractions labeled with stars were used for cryo-EM analysis. The putative HSP70 and hEag2 band is indicated. **c,** Representative electrophysiological recording from the GUV reconstituted full-length hEag2 with the voltage-pulse protocol shown underlying.

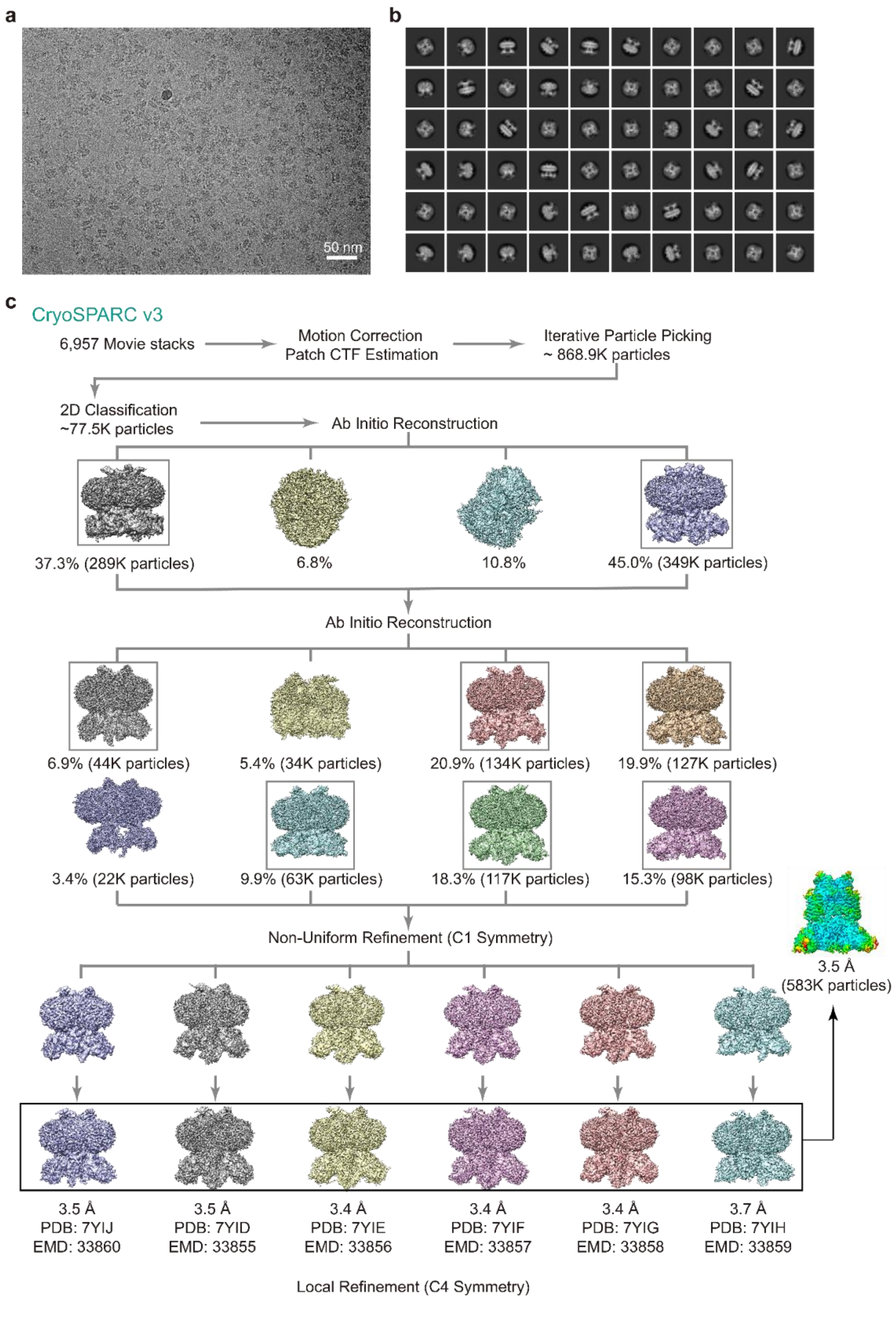

Supplementary Fig. 2 Single-particle Cryo-EM reconstructions of the hEag2 channel. a, A representative raw micrograph of the hEag2. The 50 nm scale bar is labeled in the bottom right-hand corner. **b,** Selected 2D class averages. **c,** Summary of image processing for six states of hEag2 dataset with C4 symmetry.

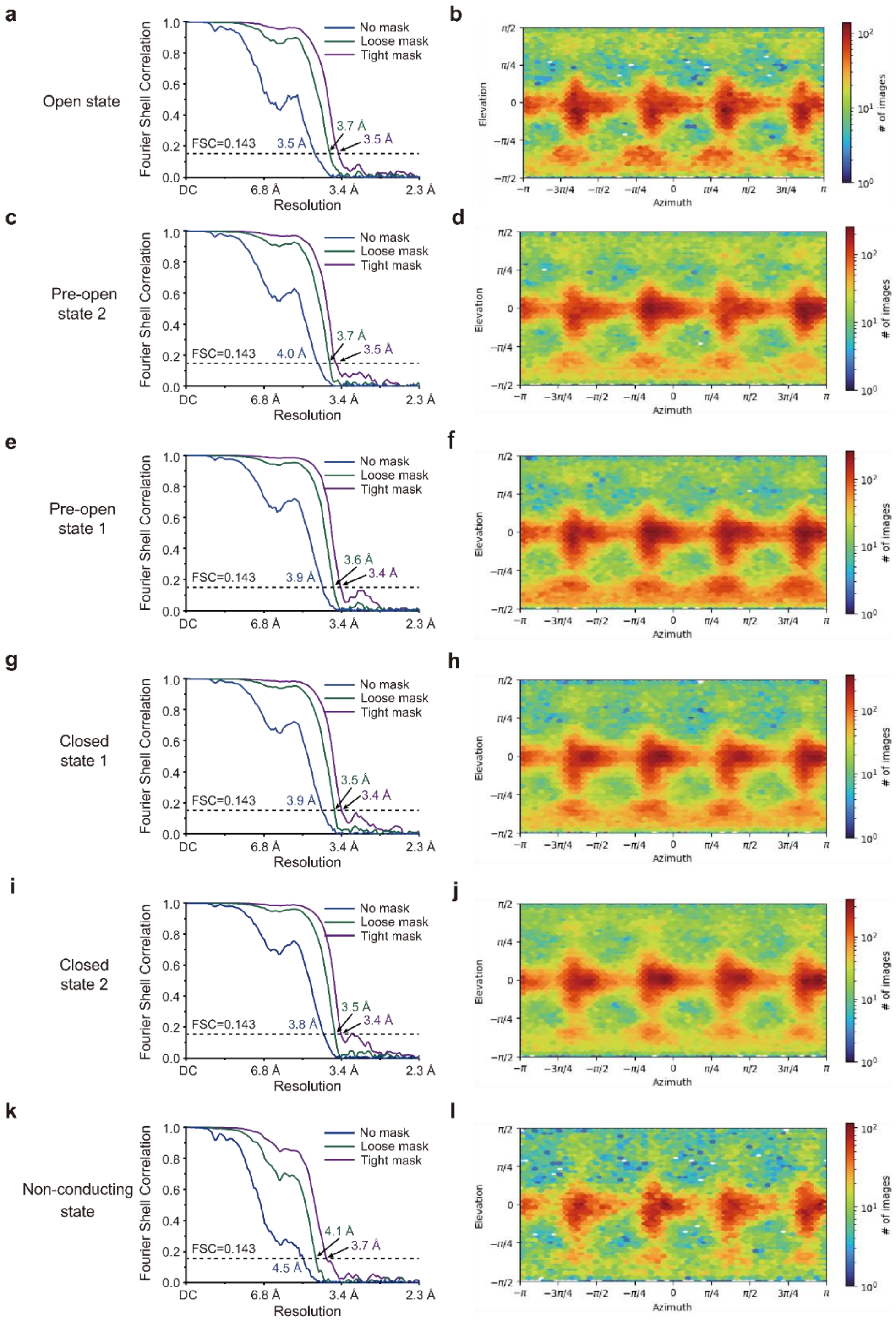

Supplementary Fig. 3 Fourier Shell correlation (FSC) curves and Euler angle distribution of particles for the final 3D reconstruction of hEag2 in six states. FSC curves between two half maps before (blue) and after loose (Green) and tight (Purple) masks of hEag2 in open state **(a)**, pre-open state 2 **(c)**, pre-open state 1 **(e)**, closed state 1 **(g)**, closed state 2 **(i)** and pore dilation but non-conducting state **(k)**, respectively. Euler angle distribution of particles for 3D reconstruction of hEag2 in open state **(b)**, pre-open state 2 **(d)**, pre-open state 1 **(f)**, closed state 1 **(h)**, closed state 2 **(j)** and pore dilation but non-conducting state **(l)**, respectively. The reported resolutions were based

on the FSC=0.143 criterion.

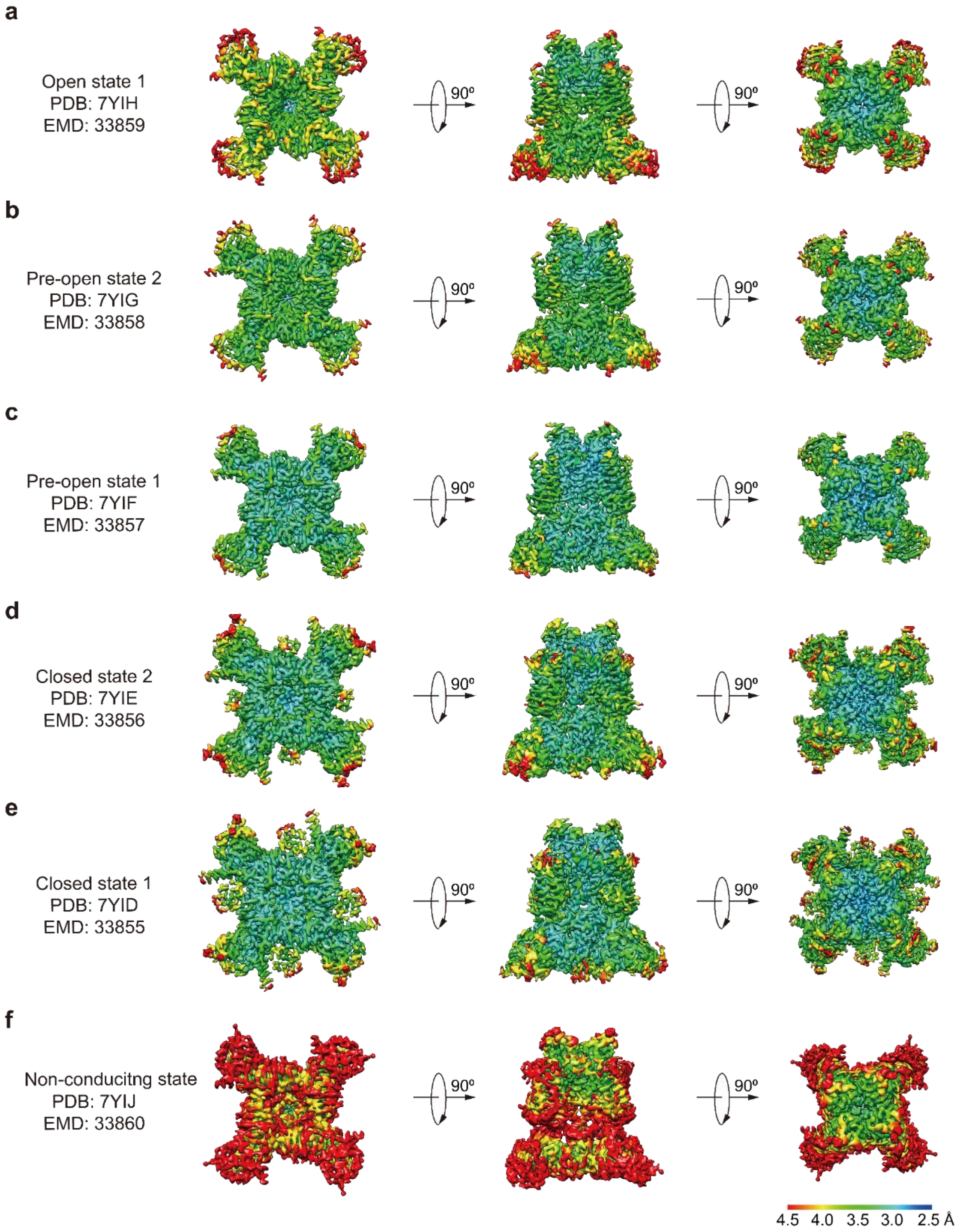

Supplementary Fig. 4 Local resolution of six density maps of hEag2 estimated by local resolution estimation in CryoSPARC. Local resolution of open state **(a)**, pre-open state 2 **(b)**, pre-open state 1 **(c)**, closed state 1 **(d)**, closed state 2 **(e)** and pore dilation but non-conducting state **(f)** hEag2 in bottom view (left), side view (middle) and top view (right) is shown.

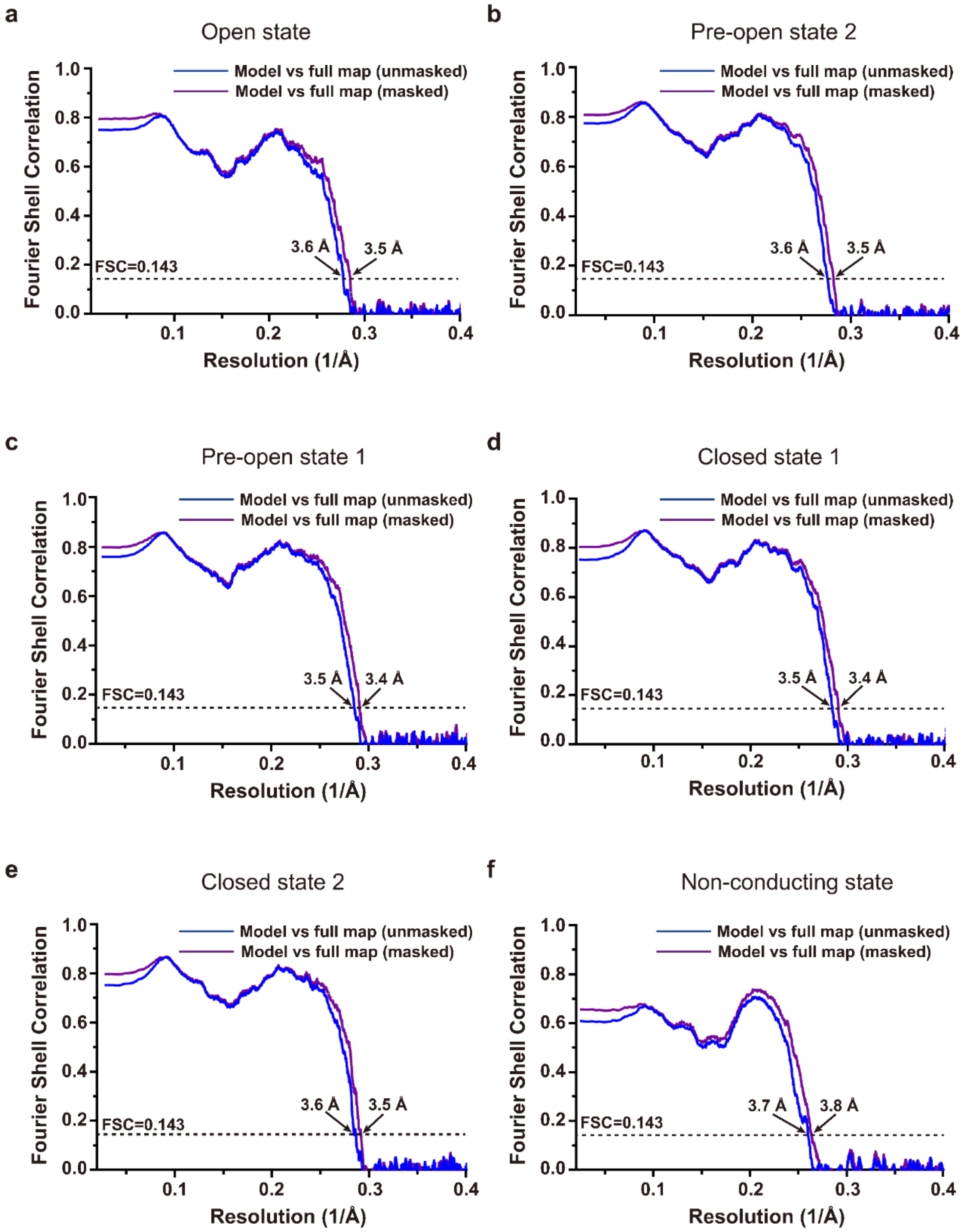

Supplementary Fig. 5 FSC curves for cross-validation: model versus unmasked full map (blue) and masked full map (purple) of hEag2 in open state **(a)**, pre-open state 2 **(b)**, pre-open state 1 **(c)**, closed state 1 **(d)**, closed state 2 **(e)** and pore dilation but non-conducting state **(f)**.

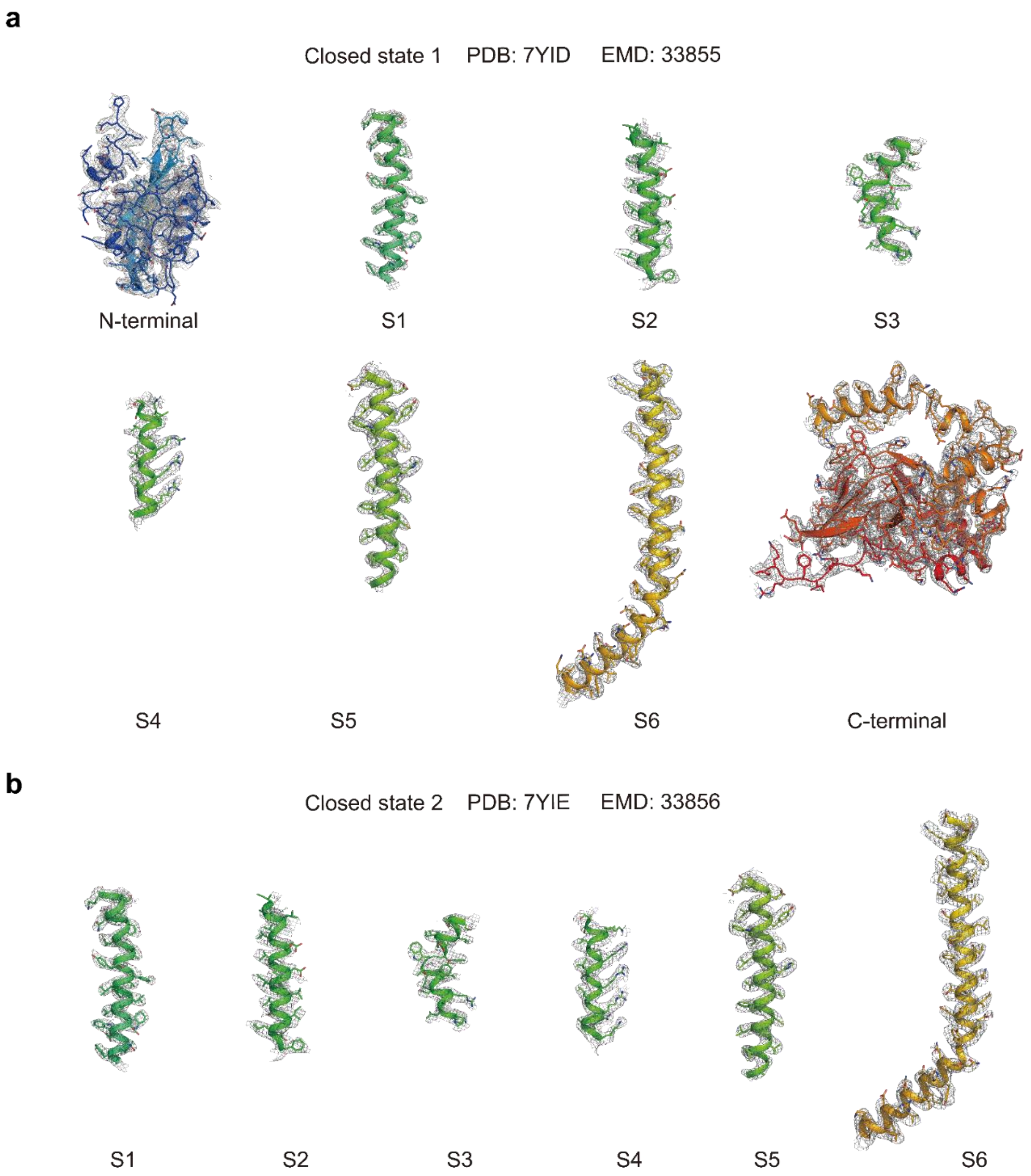

Supplementary Fig. 6 EM density of hEag2 in closed state. EM density of hEag2 in closed state 1 **(a)** and closed state 2 **(b)**.

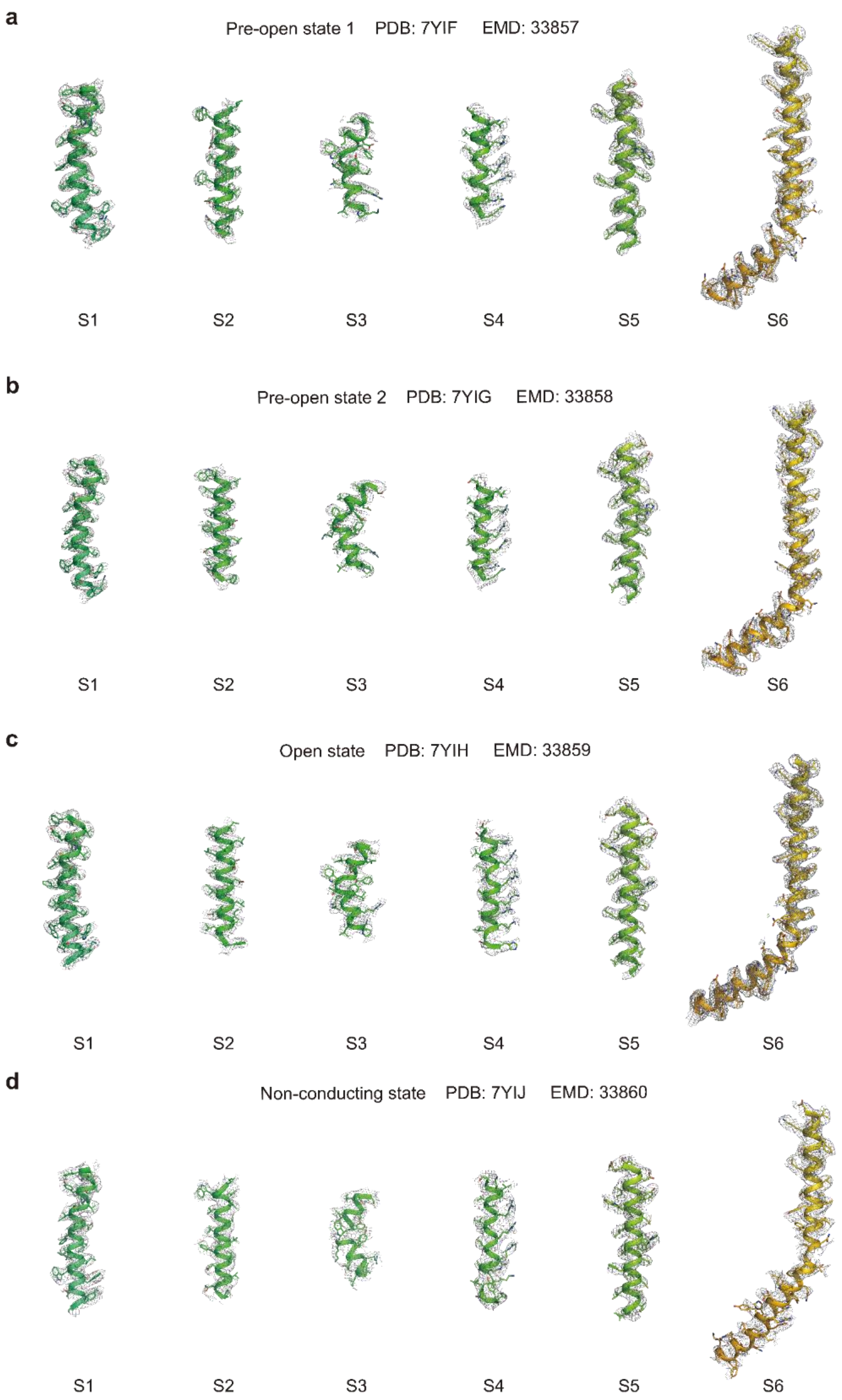

Supplementary Fig. 7 EM density of hEag2 in pre-open, open and pore dilation but non-conducting states. S1-S6 EM density of hEag2 in pre-open state 1 **(a)**, pre-open state 2 **(b)**, open state **(c)** and pore dilation but non-conducting state **(d)**.

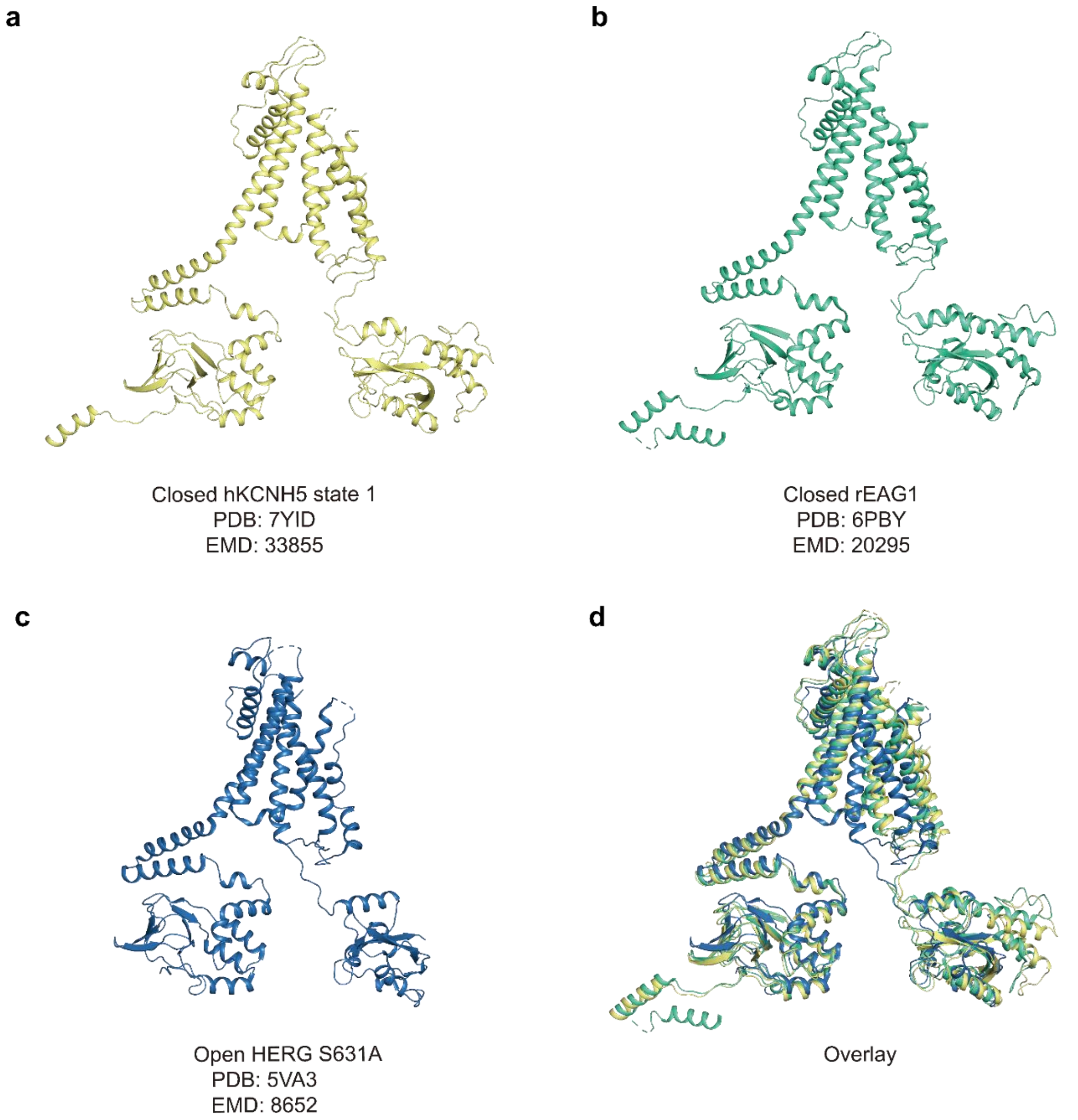

Supplementary Fig. 8 Structure comparison of hEag2, rat Eag1 and human Erg1. Subunit structure of closed hEag2 **(a)**, closed rat Eag1 **(b)** and open human Erg1 **(c)**. **(D)** Subunit structure alignment of hEag2 (yellow), rat Eag1 (green) and human Erg1 (blue).

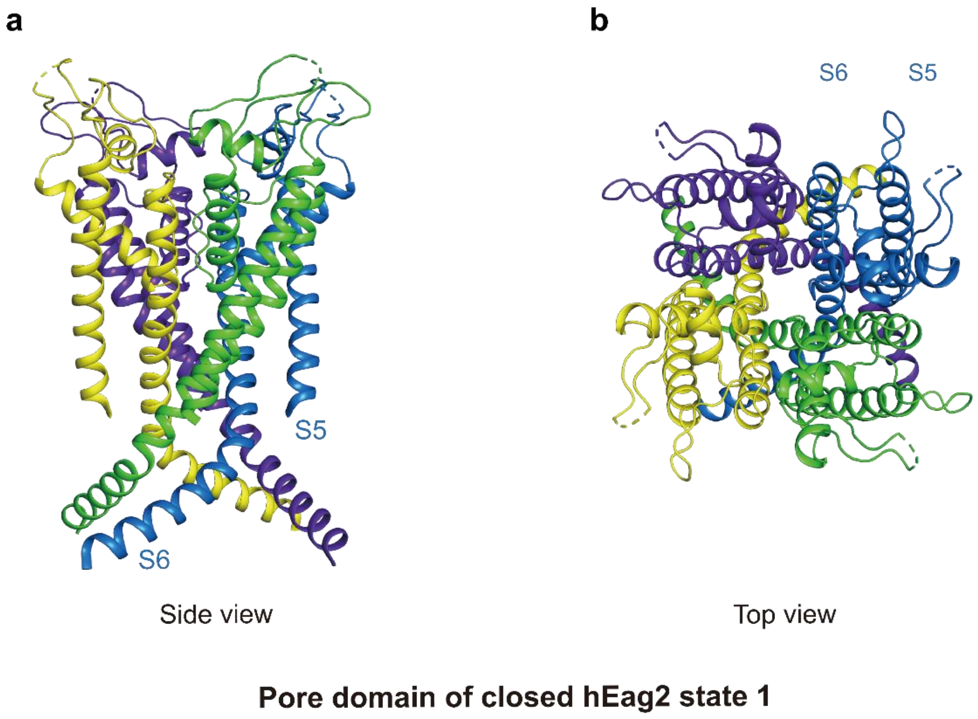

Supplementary Fig. 9. Pore domain of hEag2 in the closed state. Pore domain of closed hEag2 in side view **(a)** and in top view **(b)** was depicted as a cartoon. Each subunit was colored. S5 and S6 helices were labeled.

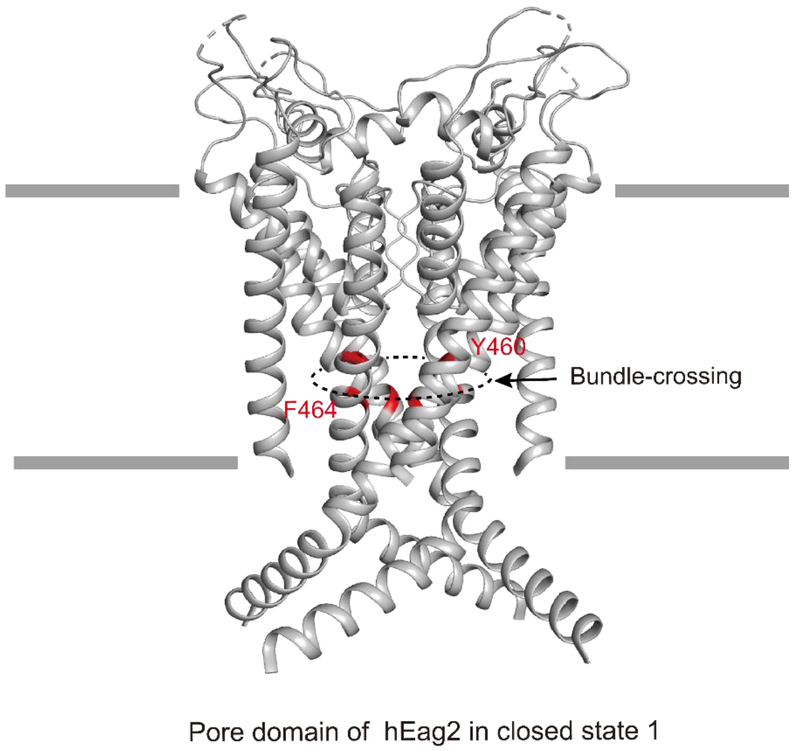

Supplementary Fig. 10 Bundle-crossing site of hEag2 in the closed state. Pore domain is shown as cartoon and colored in grey. Bundle-crossing site is labeled as a

circle with the dotted line and key residues at the bundle-crossing site, Y460 and F464, were colored in red.

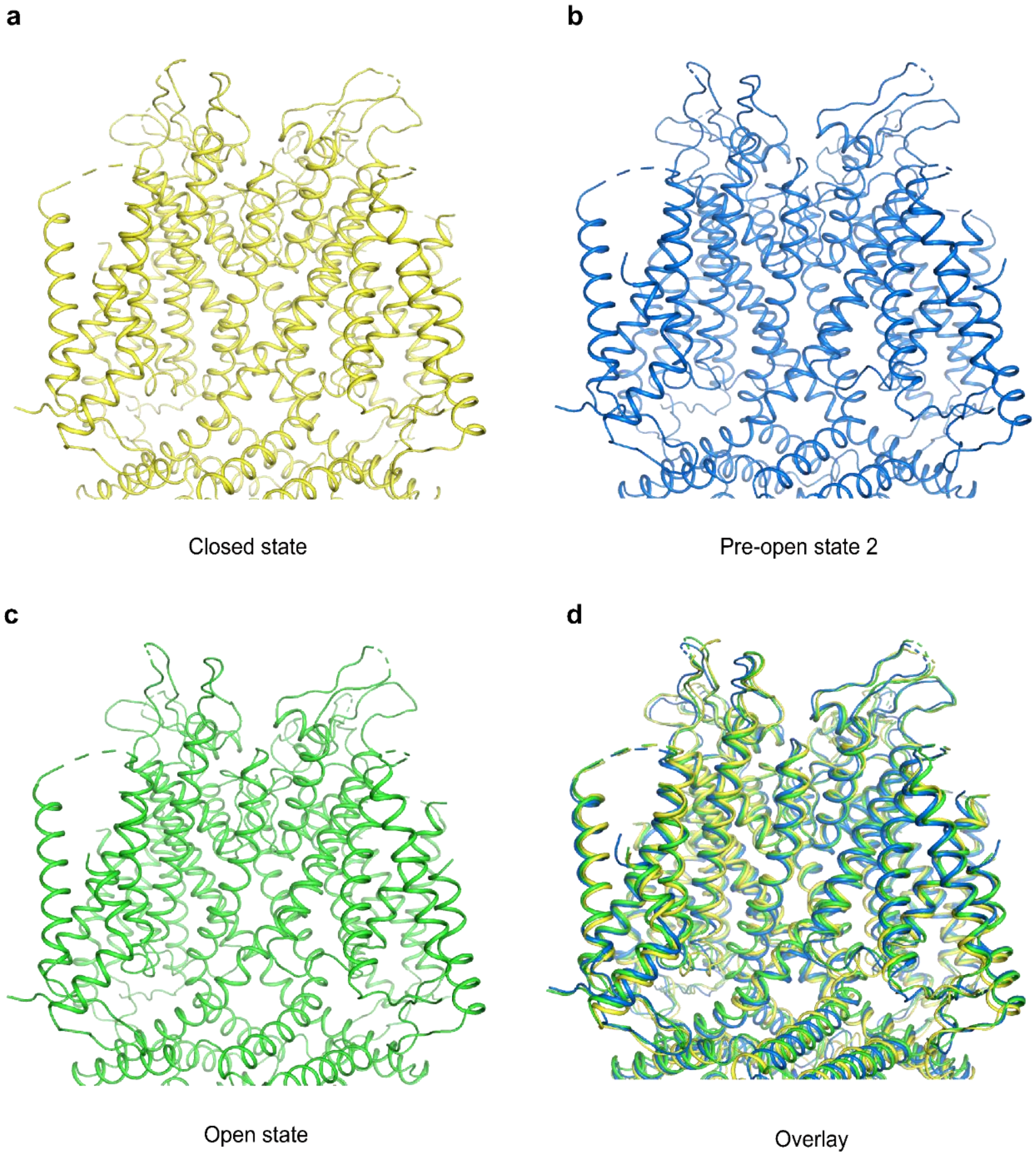

Supplementary Fig. 11 Structure comparison of extracellular ion conduction pore of hEag2 in the different states. Side view of extracellular ion conduction pore of hEag2 in the closed state (**a**, in yellow), pre-open state 2 (**b**, in blue), open state (**c**, in green) and overlay (**d**) is shown as a cartoon. The extracellular ion conduction pore of hEag2 in all the states have barely any differences.

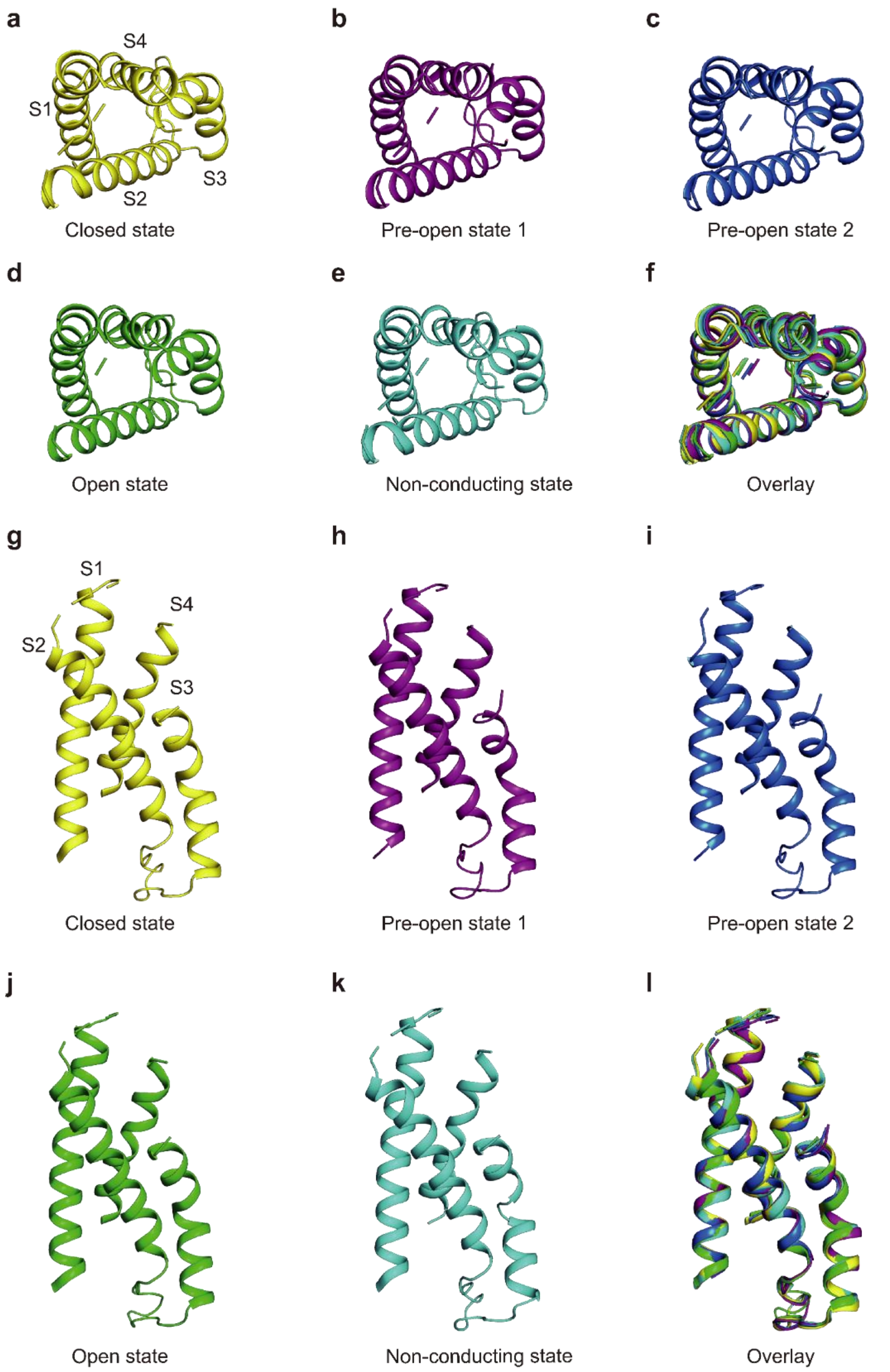

Supplementary Fig. 12 Structure comparison of hEag2 VSD during voltage activation process. Top view and side view of closed state **(a, g)**, pre-open state 1 **(b, h)**, pre-open state 2 **(c, i)**, open state **(d, j)**, pore dilation but non-conducting state **(e, k)** and the superposition of all the states were colored and shown as cartoon. S1-S4 helices were labeled. **(f, l)** are the overlay views of all states.

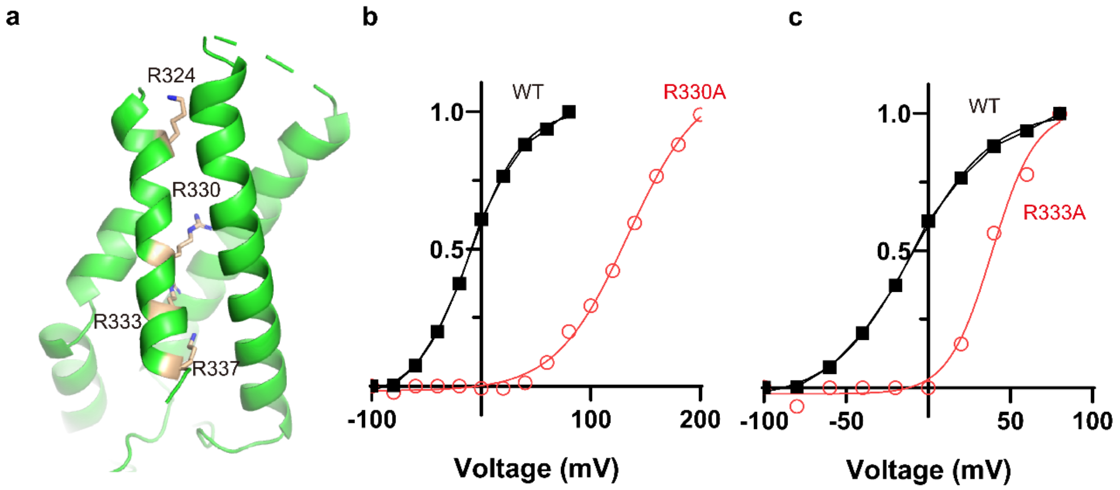

Supplementary Fig. 13 Electrophysiological analysis of VSD region. Key residues in the VSD domain **(a)**. Normalized tail currents ($(I-I_{min})/(I_{max}-I_{min})$) versus voltage (I-V plot) from wild type, R330A and R333A **(b-c)**.

	Closed hKCNH5 state 1 (PDB 7YID) (EMD 33855)	Closed hKCNH5 State 2 (PDB 7YIE) (EMD 33856)	Pre-open hKCNH5 state 1 (PDB 7YIF) (EMD 33857)	Pre-open hKCNH5 state 2 (PDB 7YID) (EMD 33855)	Open hKCNH5 (PDB 7YIH) (EMD 33859)	Pore dilation but non-conducting hKCNH5 (PDB 7YIJ) (EMD 33860)
**Data collection and processing**						
Microscope			FEI Titan Krios			
Magnification			105,000			
Voltage (KV)			300			
Detector			Gatan K3			
Electron exposure (e^-/ Å^2)			60			
Defocus range (μm)			-1.5 to -2.0			
Pixel size (Å)			0.849			
Symmetry imposed			C4			
Initial particle images (no.)			868,893			
Final particle images (no.)	133,570	126,938	116,577	97,894	63,386	44,152
Map resolution (Å)	3.4	3.4	3.4	3.5	3.5	3.7
FSC threshold	0.143	0.143	0.143	0.143	0.143	0.143
**Refinement**						
Initial model used (PDB code)	6PBY	6PBY	6PBY	6PBY	6PBY	6PBY
Model resolution (Å)	3.4	3.4	3.4	3.5	3.5	3.8
FSC threshold	0.143	0.143	0.143	0.143	0.143	0.143
Map sharpening *B* factor (Å^2)	130.7	129.3	123.7	118.9	108.9	122.9
Model composition						
Non-hydrogen atoms	21218	21222	21218	21216	21217	21209
Protein residues	2628	2628	2628	2628	2628	2628
Water	4	8	4	4	4	0
Ions	6	6	6	4	5	1
B factors						
Protein	34.09	33.32	26.56	36.48	48.95	180.45
Ligand	24.64	21.64	20.38	24.34	40.96	127.02
Water	17.82	13.21	6.90	14.06	13.23	
R.m.s. deviations						
Bond lengths (Å)	0.003	0.002	0.003	0.003	0.004	0.004
Bond angles (°)	0.754	0.539	0.758	0.753	0.958	1.034
Valiation						
MolProbity score	1.94	1.76	1.92	1.98	2.41	2.50
Clash score	8.13	6.25	7.47	9.12	17.2	21.13
Poor rotamers (%)	0.00	0.00	0.04	0.00	0.17	0.00
Ramachandran plot						
Favored (%)	91.72	93.68	91.37	91.64	84.05	83.59
Allowed (%)	7.97	6.16	8.32	8.2	14.71	15.06
Disallowed (%)	0.31	0.15	0.31	0.15	1.23	1.35

Supplementary Table. 1 Cryo-EM data collection, refinement and validation

statistics of six states of hEag2.

	$V_{0.5}$	k	Zg	DG0	n
WT	-10.0	56	0.45	-0.105	21
T468A	-6.659	42	0.61	-0.095	8
Q472R	0.3806	60	0.42	0.004	9
K337A	-98	63	0.41	-0.928 (*)	8
K324A			LOF		3
R330A	134.1	76	0.33 (*)	1.042 (*)	4
R333A	38.88	31	0.83 (*)	0.747 (*)	5

Supplementary Table. 2 Summary of EP data in this study. $V_{0.5}$ and k are half-maximum activation voltage and slope factor of the simple Boltzmann fit to activation curves; normalized tail current 1/[1+exp((V0.5-Vt)/k)]. Zg is gating charge estimated from the mean value of slope factor from the Boltzmann fit (k), as Zg=k(RT/F), where RT/F 25 mV. DG0 is calculated as RT ($V_{0.5}$/k), where RT=0.592 kcal/mol, $V_{0.5}$ and k are from Boltzmann fit. Star (*) indicate that one-way ANOVA, P <0.01 comparing to wild type hEag2.
