# Supplementary material for: Mechanism underlying delayed rectifying in human voltage-mediated activation Eag2 channel
Source: Nat Commun. 2023 Mar 16;14:1470. doi: 10.1038/s41467-023-37204-6 (PMC10020445; doi:10.1038/s41467-023-37204-6)
Supplement: Supplementary file 3 — Description of Additional Supplementary Files [file 41467_2023_37204_MOESM3_ESM.pdf]

Description of Additional Supplementary Files

File Name: Supplementary Movie 1
Description: Voltage activation mechanism of hEag2 channel. The iris like rotation of hEag2 during voltage dependent activation.
